# Improved Loading Capacity and Viability of Probiotics Encapsulated in Alginate Hydrogel Beads by In Situ Cultivation Method

**DOI:** 10.3390/foods12112256

**Published:** 2023-06-03

**Authors:** Yachun Huang, Lin Zhang, Jielun Hu, Huan Liu

**Affiliations:** 1 State Key Laboratory of Food Science and Resources, China-Canada Joint Laboratory of Food Science and Technology (Nanchang), Key Laboratory of Bioactive Polysaccharides of Jiangxi Province, Nanchang University, Nanchang 330047, China; 402313320010@email.ncu.edu.cn (Y.H.); hujielun2005@hotmail.com (J.H.); 2 Microbiota I-Center (MagIC), The Chinese University of Hong Kong, Hong Kong SAR, China; 3 Department of Medicine and Therapeutics, Faculty of Medicine, The Chinese University of Hong Kong, Hong Kong SAR, China; 4 Centre for Gut Microbiota Research, Faculty of Medicine, The Chinese University of Hong Kong, Hong Kong SAR, China

**Keywords:** microencapsulation, probiotic, alginate, hydrogel beads, in situ cultivation

## Abstract

The objective of this research was to encapsulate probiotics by alginate hydrogel beads based on an in situ cultivation method and investigate the influences on the cell loading capacity, surface and internal structure of hydrogel beads and in vitro gastrointestinal digestion property of cells. Hydrogel beads were prepared by extrusion and cultured in MRS broth to allow probiotics to grow inside. Up to 10.34 ± 0.02 Log CFU/g of viable cell concentration was obtained after 24 h of in situ cultivation, which broke through the bottleneck of low viable cell counts in the traditional extrusion method. Morphology and rheological analyses showed that the structure of the eventually formed probiotic hydrogel beads can be loosed by the existence of hydrogen bond interaction with water molecules and the internal growth of probiotic microcolonies, while it can be tightened by the acids metabolized by the probiotic bacteria during cultivation. In vitro gastrointestinal digestion analysis showed that great improvement with only 1.09 Log CFU/g of loss in viable cells was found after the entire 6 h of digestion. In conclusion, the current study demonstrated that probiotic microcapsules fabricated by in situ cultivation method have the advantages of both high loading capacity of encapsulated viable cells and good protection during gastrointestinal digestion.

## 1. Introduction

Probiotics have multiple health benefits for humans and are used not only as health supplements but also for the prevention and treatment of diseases such as diarrhea, colon cancer, obesity, diabetes, and inflammation [1]. However, the harsh gastrointestinal environment affects the ability of probiotics to perform their physiological activity. Microencapsulation of probiotics is an effective and feasible method to enhance their stability and cell survival. In addition to improving the survival rate of probiotics during gastrointestinal digestion, microencapsulation technology can also promote the stability of probiotics by preventing adverse reactions with external factors and ensuring controlled and targeted release in the gastrointestinal tract [2].

Extrusion is one of the most commonly used technologies to prepare probiotic microcapsules, where the microcapsules are formed by adding the probiotic to the wall solution and dropping it into the curing solution as droplets (using a nozzle) to form tiny particles. The extrusion method exhibits numerous advantages, such as simplicity, low cost, mild process conditions, uniform particle size, and high cell viability. For instance, Marluci et al. [3] used co-extrusion technology with the wall materials of alginate and shellac to prepare probiotic (*Lactobacillus paracasei* BGP-1) microcapsules and found that the formulation produced with alginate–shellac and coconut fat can reduce the porosity of microcapsules and was the most effective in improving probiotic survival in simulated gastrointestinal fluids, in which 7.5 Log CFU/g of probiotics survived at the end of gastrointestinal digestion. Khan et al. [4] developed legume protein isolate-based microcapsules with alginate using the extrusion method and found that the protection of entrapped *Bifidobacterium adolescentum* was significantly improved over the pH-challenge test with end-point cell reduction values of 2.0–2.6 Log CFU/g of capsule compared to that of 4.5 Log CFU/g in free cells. Sandoval et al. [5] utilized alginate and pectin as wall materials to entrap *Lactobacillus casei* using the extrusion method for application in yogurt and found that the binary calcium–alginate–pectinate beads matrices provided good protection against bile salts.

Recently, probiotic hydrogel beads fabricated by inoculating cells into blank hydrogel beads and then in situ co-cultured has caught the attention of researchers and has been applied in the area of reusable heavy metal ions biosorbents, biofilm, and bacterial cellulose nanocomposite beads. For instance, Park et al. [6] inoculated *zoogloea ramigera* (KCTC 2582) cells into hydrogel beads consisting of sodium alginate and found that the specific uptake capacity of cadmium was 1.45 mg/g of the crosslinked capsule biosorbent, which can maintain the mechanical strength and adsorption/desorption capacity even after 30 cycles of repeated uses. Heumann et al. [7] inoculated *Lacticaseibacillus paracasei* (ATCC334) into hydrogel beads consisting of low methoxyl pectin and found that the cells encased in these pectinate beads exhibited increased resistance to acidic stress (pH 1.5), osmotic stress (4.5 M NaCl), and freeze-drying processes. Kim et al. [8] inoculated *Gluconacetobacter xylinus* into alginate beads and with 36 h of cultivation; the entire bead surface was covered with cellulose fibers (~30 nm), which was metabolized by the cells. After immobilizing lipase into this kind alginate/bacterial cellulose beads, the enzyme activity and specific activity were 2.6- and 3.8-fold higher than that of lipase immobilized in cellulose beads.

Although in situ cultivation technology has shown its superiority regarding protection of bioactive core materials to the outside adverse environments, few studies have been performed as to whether the application of this technology could improve the gastrointestinal target delivery property of probiotic bacteria, which gives us a chance to further move on. Thus, the aim of this study was to encapsulate probiotic bacteria through in situ cultivation technology and analyze its effects on the loading capacity, surface and internal structure, viscoelasticity, as well as the gastrointestinal properties of probiotic hydrogel beads and the corresponding mechanisms.

## 2. Materials and Methods

### 2.1. Materials

*Lactobacillus plantarum* 550 was purchased from Sichuan Gaofuji Biological Technology Co., Ltd. (Chengdu, China). Sodium alginate is pharmaceutical grade, purchased from Aladdin Biochemical Technology Co., Ltd. (Shanghai, China), calcium chloride (AR, Xilong Science, Guangzhou, China); pepsin (Solarbio, Beijing, China, viability > 250 u/mg); pancreatic protease (Solarbio, China, viability > 250 u/mg); porcine bile salt (Macklin, Shanghai, China).

### 2.2. Hydrogel Beads Preparation

Dissolve the appropriate amount of sodium alginate powder in water and stir with a cantilever stirrer for about 4 h to allow complete hydration. Dried anhydrous CaCl_2_ powders were added to the solution and stirred until completely dissolved. All instruments and reagents were sterilized using an autoclave at 121 °C for 15 min prior to sample preparation.

Bacterial cells of *L. plantarum* 550 were harvested by centrifugation at 4000 rpm for 10 min. Then, *L. plantarum* 550 cells were dispersed (6 LogCFU/g) in the alginate solution until a uniform suspension was obtained. The cell suspension was dispersed dropwise using a syringe auto-pump into sterile CaCl_2_ (1 %wt) as a hardening solution at the rate of 1 mL/min in order to produce small beads. After a gelation time of 10 min in CaCl_2_, the beads containing *L. plantarum* 550 were filtered through a 200-mesh gauze filter and washed with bacteria-free water 2–3 times. Finally, the surface of the hydrogel beads was blotted with bacteria-free paper to obtain the sample of hydrogel beads containing about 6 LogCFU/g *L. plantarum* 550 called before incubation, containing a low concentration of the bacteria group (BLP).

In order to describe and compare the changes in the apparent morphology, internal structure, and resistance to external damage of the hydrogel bead samples during the incubation process, the hydrogel bead samples without *L. plantarum* 550 (BC) and the control group (CLP) that contained a high concentration of *L. plantarum* 550 (about 9 LogCFU/g) were prepared in the same way.

### 2.3. Hydrogel Beads Post-Encapsulation Cultivation

The sample named BLP containing approximately 6 LogCFU/g *L. plantarum* 550 and the sample named BC without bacteria were put into the appropriate amount of MRS broth and incubated in a constant temperature incubator at 37 °C for a period of time.

After 24 h of incubation, the samples were filtered through a 200-mesh gauze filter and washed with bacteria-free water 2–3 times, and finally the surface of the hydrogel beads was blotted with bacteria-free paper to collect the after-incubation bacteria-containing group (ALP) obtained from the cultivation of BLP containing about 6 LogCFU/g *L. plantarum* 550 and the after-incubation non-bacteria group (AC) samples obtained from the cultivation of BC samples without bacteria, respectively. The composition and preparation of each hydrogel bead sample are shown in Table 1.

The pH of the external MRS broth was measured at different times during the incubation process, while a certain quantity of hydrogel beads containing the bacteria was taken in a previously prepared PBS solution and dispersed in a high-speed disperser at 4000 rpm for about 10 min to fully release the *L. plantarum* 550 encapsulated in the hydrogel beads, and the plates were spread to count the viable bacteria.

### 2.4. Particle Size Measurement

Ten hydrogel beads were randomly selected from each group and their diameters were measured using a micrometer. The mean diameter ± standard deviation was calculated, as well as the percentage change between corresponding groups before and after cultivation.

### 2.5. Microscopic and SEM Observation

Each group of hydrogel beads was placed entirely on a slide, and a microscope was used to observe the whole and partial position of the hydrogel beads; magnification was amplified at 50–400×. The observation was repeated 3 or more times.

The hydrogel beads were first pre-frozen in a −80 °C refrigerator for 4 h, followed by freeze-drying in a vertical freeze-dryer (Labconco, Kansas City, MO, USA) at 0.025 mbar vacuum and −55 °C for 72 h. The freeze-dried beads were split in half, then the surface of the complete freeze-dried beads and the cross-section of the freeze-dried beads were sprayed with gold. Finally, the surface and cross-section microstructure of the beads were observed by field emission scanning electron microscope (Hitachi SU8010, Tokyo, Japan). The observation was repeated 3 or more times.

### 2.6. Rheology Experiments

The method of Dai et al. [9] was used, with a slight modification as follows. Hydrogel beads of each group were individually exposed to dynamic strain sweep and frequency sweep experiments on a TA ARES-G2 strain-controlled rheometer with a standard parallel plate (25 mm diameter). A layer of hydrogel beads was dispersed on the plate, and the beads were carefully placed on the plate to minimize the gap between samples. Strain sweep measurements were performed from 0.00126% to 10% to determine the linear viscoelastic region (LVR) at 25 °C and a constant angular frequency of 10 rad/s. A constant strain of 0.01% was selected in the LVR for frequency sweep experiments in the frequency range of 10–0.1 Hz, the elastic modulus (storage modulus, G′) and viscous modulus (loss modulus, G″) were recorded to provide insight into the hydrogel network structure and viscoelastic behavior of hydrogel beads. The experiment was repeated four times.

η′ is called the dynamic viscosity and is the energy dissipation part of the complex viscosity, which is related to the loss modulus and indicates the contribution of viscosity. η′ is calculated by the following Equation (1):

η′ = so/iωgo × sinδ = G″/ω,(1)

η″ is called the imaginary viscosity and is a measure of elasticity and energy storage, which is related to the dynamic modulus and indicates the contribution of elasticity. η″ is calculated by the following Equation (2):

η″ = so/iωgo × cosδ = G′/ω,(2)

The utilization of dynamic viscosity and imaginary viscosity can effectively characterize the viscoelastic properties of polymer systems.

### 2.7. In Vitro Simulation of Gastro-Intestinal Digestion

Firstly, the preparation of simulated gastric solution and simulated intestinal solution was carried out. The gastric solution consisted of 0.32 g of pepsin and 0.2 g of NaCl in 100 g of ultrapure water, mixed well, and then the pH of the solution was adjusted to 2.0 with 1 M HCl. The intestinal solution included 0.1 g of trypsin and 0.08 g of pig bile salt in 100 g of 0.2 M PBS solution, mixed well. Then, the simulated intestinal solution and simulated gastric solution were incubated in 37 °C water for 20 min and then passed through a filter membrane with a pore size of 0.22 μm, respectively, for sterilization.

Then, 1 g of hydrogel beads or 1 mL of probiotic suspension with different concentrations of ALP and CLP were added to 8 centrifuge tubes containing 9 mL of simulated gastric solution, respectively, and mixed with constant vibration at 180 rpm in a shaker at 37 °C. At 30, 60, 90, and 120 min of incubation time, 4 centrifuge tubes were taken and 10 mL of 0.2 M PBS solution was added immediately, and the solution in each tube was decapsulated at 4000 rpm for 10 min in a high-speed disperser to release *L. plantarum* 550 from the hydrogel beads, and the solution in each of the 4 tubes was diluted in a gradient and counted using plate coating. Immediately after 120 min of simulated gastric solution incubation, 10 mL of simulated intestinal solution was added to each of the other 4 centrifuge tubes, which were also shaken and mixed continuously at 180 rpm in a 37 °C shaker, and the solutions in these 4 centrifuge tubes were diluted in a gradient at 1, 2, 3, and 4 h of incubation time, respectively; additionally, the viable bacteria were counted using a plate.

### 2.8. Statistical Analysis and Figure Plotting

All measurements were carried out in triplicate. The data were expressed as mean ± standard deviation and subjected to analysis of variance (ANOVA). Statistical analysis was performed with SPSS Statistics Software 24.0. Differences were considered significant when *p* < 0.05. Origin 2018(Version 9.0) was applied to form figures.

## 3. Results and Discussion

### 3.1. Growth Characteristics of Bacteria Encapsulated in Hydrogel Beads during Incubation

**Figure 1:**
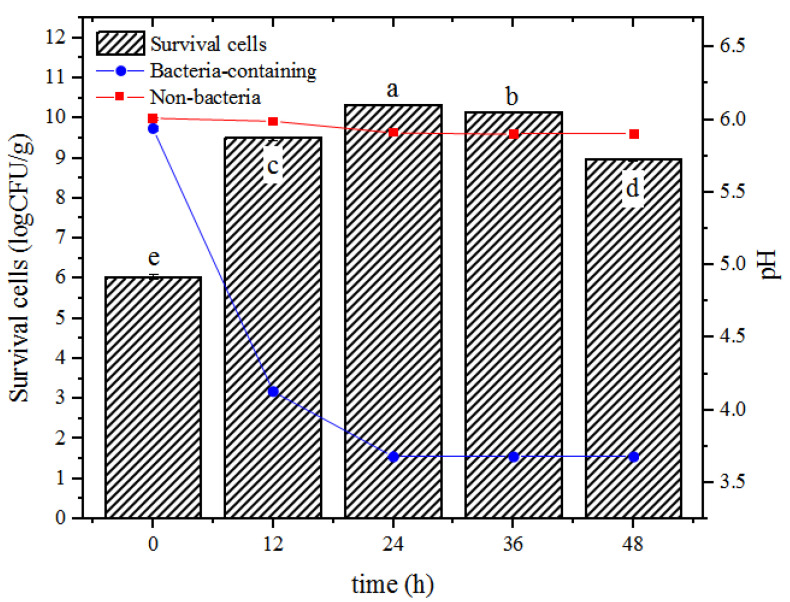
Variations in the number of survival cells inside hydrogel beads (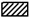) and pH of external MRS broth during incubation with (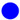) or without (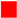) inoculation of probiotic bacteria. Columns with different letters (a–e) are significantly different (*p* < 0.05).

Figure 1 showed the variations in viable bacterial counts inside hydrogel beads and the pH of external growth medium while culturing the microcapsules. As the incubation time increased, the bacterial counts inside the hydrogel beads first increased and then decreased. The fastest increase rate was observed within the first 12 h of incubation, and the highest counts were obtained within 24 h; the counts gradually declined afterward. The pH of the external solution also varied with the bacterial counts; the values rapidly decreased within the first 24 h of incubation and then stabilized. Notably, these variations in viable cell count and pH corresponded well with the bacterial growth curve, where the logarithmic growth phase was observed from 0 to 12 h, the stationary phase was observed from 12 to 36 h, and the decline phase was observed after 36 h. These observations indicated that the growth of *L. plantarum* 550 was optimal after encapsulation and subsequent culturing in the MRS medium, and the three-dimensional hydrogel network of beads did not inhibit bacterial growth and metabolism, which could be attributed to the porous structure inside the calcium alginate hydrogel beads and the channels that existed on the surface [10]. On one hand, the porous structure of the hydrogel beads provided spaces for the propagation of probiotic cells [11]. On the other hand, the surface channels allowed MRS broth to enter inside the beads, thereby providing nutrients to the encapsulated bacteria at early stages and allowing diffusion of metabolites into the external medium at later stages.

In the conventional method, the maximum viable bacterial count in sodium alginate microspheres was approximately 7 Log CFU/mL. For instance, Ding et al. [12] incorporated carboxymethyl konjac glucomannan–chitosan complex double emulsion nanogels into alginate hydrogel beads for the encapsulation and delivery of *L. roxellanae*. The author observed that the viable counts in the hydrogel beads before and after lyophilization were around 10^7^–10^8^ Log CFU/mL and 10^6^–10^7^ Log CFU/mL, respectively; Mirmazloum et al. [13] co-encapsulated probiotic *L. acidophilus* and *Ganoderma lucidum* (medicinal mushroom) extracts within calcium alginate beads and determined the viable counts inside of approximately 7 Log CFU/mL. Exhilaratingly, in our study, viable bacterial counts up to 10.34 ± 0.02 Log CFU/g of hydrogel beads were attained and were around 1000 times higher than those obtained with the traditional method, which broke through the bottleneck of low viable cell counts in traditional alginate beads.

### 3.2. Particle Size Variations of Hydrogel Beads during Incubation

Table 2 listed the changes in the particle size of microcapsules with or without bacteria before and after culturing. With the sodium alginate concentrations increased, the particle sizes of the microcapsules in both non-bacteria and bacteria-containing groups enlarged. Firstly, at higher concentrations, more mannuronic acid (M) will participate in lateral association, and more guluronic acid (G) will chelate with Ca^2+^ to form more stable “egg box” type structures, leading to better sphericity of the hydrogel beads. Moreover, an increase in sodium alginate concentration also increased the viscosity of the extrusion solution leading to the occurrence of severe entanglement between the alginate molecular chains and finally resulting in the formation of hydrogel beads with larger particle sizes [14]. The particle sizes of hydrogel beads post-incubation were larger than those of hydrogel beads pre-incubation, which can be attributed to the swelling properties of hydrogel beads. Water molecules have the ability to weaken the internal hydrogen bonds of carbohydrates, which strongly affects the conformation in aqueous solution [15]. As reported by Plazinski [16], through the molecular dynamics (MD) simulation analysis, the ability of alginate combining with calcium ions showed a reducing trend with the existence of hydrogen bonds. Thus, when the hydrogel beads were exposed to water during incubation in MRS broth, the water-mediated hydrogen bonding enlarged the distance between the ionic cross-linked blocks, leading to the loosening of the hydrogel structure.

**Figure 2:**
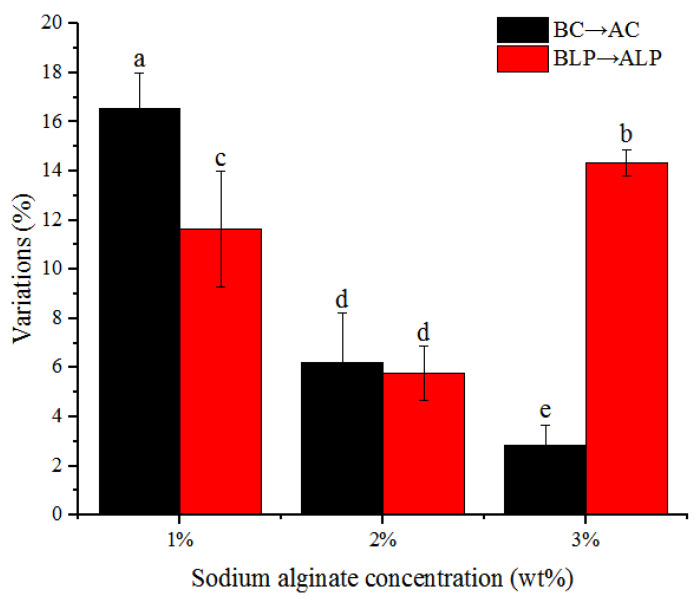
Particle size variation rates of no-bacteria and bacteria-containing beads before and after 24 h incubation. “BLP → ALP” and “BC → AC” refers to particle size variation rates of hydrogel beads prepared at the same sodium alginate concentration without bacteria after 24 h incubation. “BLP → ALP” refers to particle size variation rates of hydrogel beads prepared at the same sodium alginate concentration with bacteria after 24 h incubation. Columns with different letters (a–e) are significantly different (*p* < 0.05).

The increasing proportions of particle size between samples with the same formula before and after incubation were shown in Figure 2. Regarding the BC and AC groups, the increasing rates of particle sizes gradually decreased with the increasing of alginate concentrations, as the hydrogel beads that were prepared with a higher concentration of alginate had a denser structure and better swelling resistance than those prepared with low alginate concentrations. Considering the change from BLP to ALP groups, the increasing rates of particle size first decreased and then increased with the increase in alginate concentrations. For bacteria-containing groups (BLP → ALP), in addition to the swelling effect, two extra factors would also influence the particle sizes of beads. Firstly, hydrogel beads were stretched with the constant growth of internal colonies. Second, lactic acid produced by bacteria would reduce the ionization degree of cross-linked polymer chains and increase the hydrogen ion electric density around the surface of hydrogel beads, and as a result, the intermolecular hydrogen bond interaction between water and calcium alginate molecular chains were greatly hindered resulting in a reduced influence of the hydrogen bond on the calcium binding ability of G-blocks, thereby decreasing the swelling degree of alginate beads [17]. For bacteria-containing microbeads with 1% alginate, due to the relatively larger internal void space within the cross-linked network, abundant space for bacterial growth can be provided, resulting in the enlargement effect induced by bacterial growth being less than the tightening effect originated by acid; therefore, the increasing rate in particle size of the bacteria-containing beads (1% BLP → ALP) was lower compared with that of the non-bacteria beads (1% BC → AC). For bacteria-containing microbeads with 3% alginate concentration, the inside structure of the microbeads was denser and provided less space for bacterial growth. Thus, the pore enlargement effect induced by bacterial growth was greater than the polymer chain-tightening effect that originated from the contact of acids; therefore, the particle size of the bacteria-containing beads (3% BLP → ALP) was higher compared with that of the non-bacteria beads (3% BC → AC). At 2% alginate concentration, the pore enlargement effect of bacterial growth was exactly equal to the tightening effect of molecular chains induced by acid; therefore, there was no significant difference between the increasing range of particle size within bacteria-containing (2% BLP → ALP) and non-bacteria beads (2% BC → AC).

### 3.3. Microstructure Variations of Hydrogel Beads during Incubation

**Figure 3:**
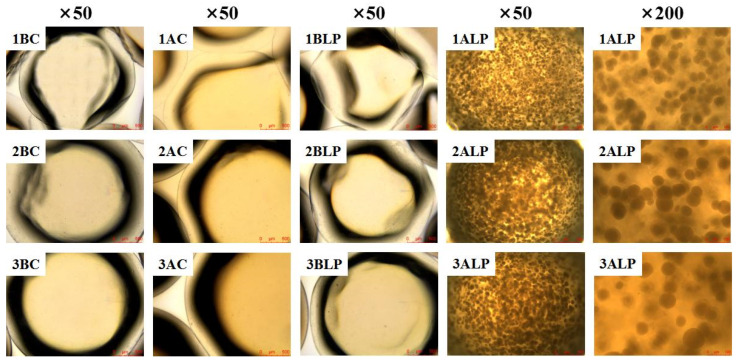
Optical microscopy observation of different hydrogel beads.

The optical microscopy images of different hydrogel beads were shown in Figure 3. Overall, we found that with the increase of alginate concentrations, the particle sizes of beads increased, corresponding with better sphericity and less trailing phenomenon. For the non-bacterial containing group, a smoother surface and better sphericity can be found in AC samples compared with that in BC samples, which could be due to the swelling effect during cultivation. For the bacteria-containing group, remarkable variation can be observed, as a mass of tiny biofilm-like aggregated bacteria microcolonies were found uniformly distributed within the cultivated hydrogel beads (BLP). Moreover, with the increase of alginate concentrations, the size of these microcolonies increased; however, the density of these colonies decreased (as observed in the same field of view). The predominant reason for the increased size in microcolonies can be due to the enhancement of molecular chain structure within the hydrogel bead, as high sodium alginate concentration would mitigate the acid-induced shrinkage (Table 2 and Figure 2) and reduce the space constraint for the bacterial colony during growth, finally leading to the augmentation of colony volume. Additionally, the proportion of sodium alginate molecules in unit volume increased with its concentration, which thus reduced the space available for bacterial growth resulting in a decline in the number of bacterial colonies per unit volume.

**Figure 4:**
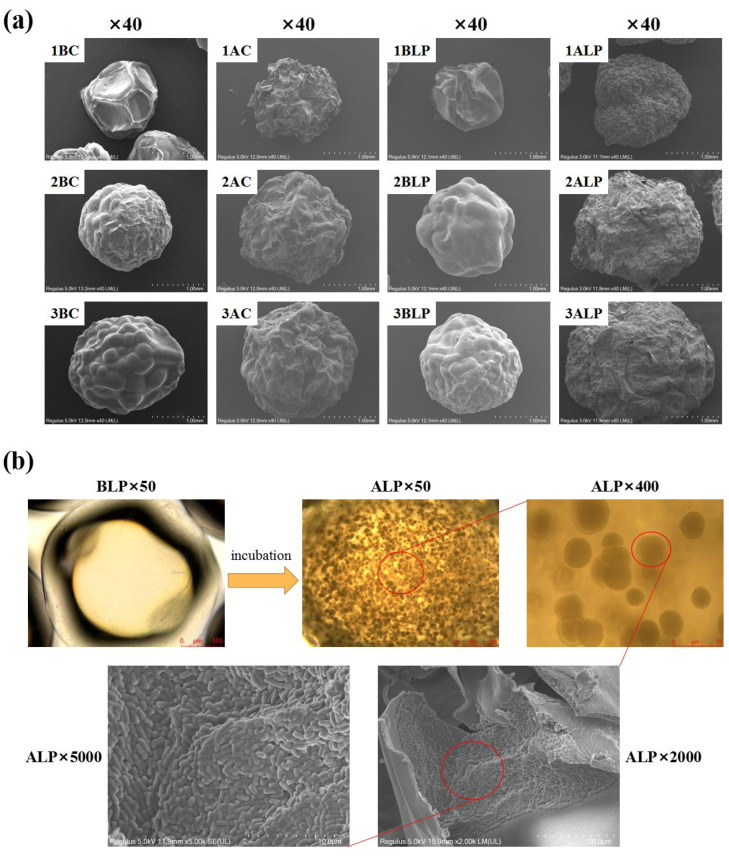
(**a**) Scanning electron microscopy (SEM) of different hydrogel beads; (**b**) schematic diagram of internal structure of ALP samples as observed by optical microscopy and SEM, respectively.

The surface morphology of lyophilized probiotic microcapsules was further observed using SEM (Figure 4a). The particle size of hydrogel beads gradually increased with an increase in sodium alginate concentrations corresponding with better spherical shape, which can also be found in Table 2 and Figure 3. Moreover, the surface of the hydrogel beads became more wrinkled after culturing, which could be due to the swelling caused by movement and subsequent deposition of sodium alginate molecules. After cutting the lyophilized ALP bead sample (2% alginate concentration) and observing the cross-section, a large number of voids inside the hydrogel beads filled with probiotic bacteria (Figure 4b) can be observed, which further confirmed that probiotic bacteria can grow optimally within the voids of alginate beads during cultivation.

### 3.4. Rheological Property Analysis

**Figure 5:**
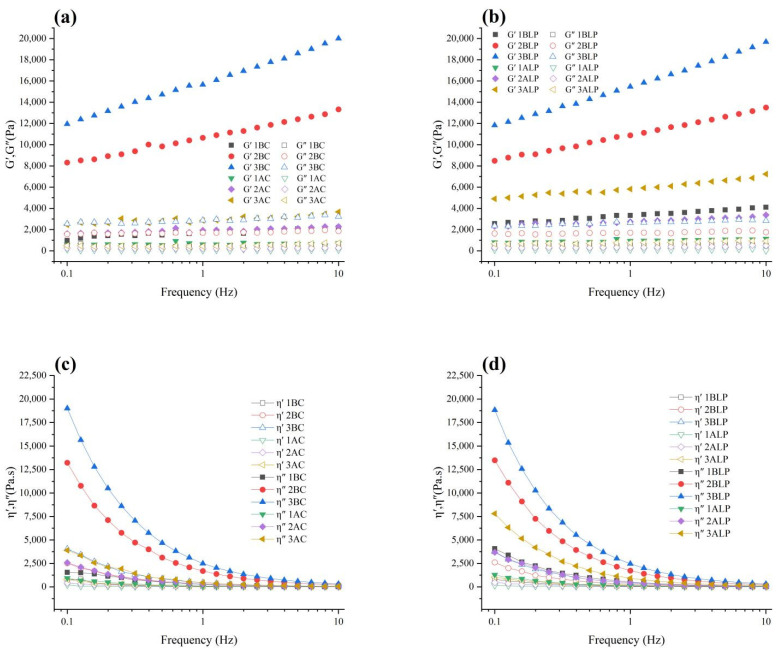
Elastic modulus (storage modulus, G′), viscous modulus (loss modulus, G″), dynamic viscosity (η′), and imaginary viscosity (η″) of different hydrogel bead samples. (**a**) the elastic modulus (storage modulus, G′) and viscous modulus (loss modulus, G″) of BC and AC; (**b**) the elastic modulus (storage modulus, G′) and viscous modulus (loss modulus, G″) of BLP and ALP; (**c**) Dynamic viscosity (η′) and imaginary viscosity (η″) of BC and AC; (**d**) Dynamic viscosity (η′) and imaginary viscosity (η″) of BLP and ALP.

Small-amplitude oscillation strain and frequency scans of hydrogel beads were performed to compare the viscoelastic variations before and after incubation (Figure 5a,b). Overall, all samples showed an increasing trend in modulus with frequency, as alginate molecular chains did not have enough time to rearrange at high frequencies and thus exhibited enhanced elasticity [18]. The storage modulus (G′) was greater than the loss modulus (G″) in all groups, indicating a relatively stable cross-linked hydrogel structure was formed in all hydrogels [19,20]. Meanwhile, for samples with the same preparation process but different alginate concentrations, G′ tended to rise with the increase of sodium alginate concentrations, which can be due to the fact that more G-blocks were involved in the crosslinking section during the hydrogel formation and the distance between crosslinks decreased leading to the limitation in the movement of alginate molecular chains and the raising in the number of rigid connections.

Comparing AC to BC and ALP to BLP samples that have the same alginate concentration, the cultured beads exhibited lower G′ than that in the uncultured samples, which can be attributed to the absorption of water and corresponding swelling. The hydrogen bonding interaction mediated by water has two effects: firstly, it weakens the internal hydrogen bonds of carbohydrates; secondly, it reduces the ability of alginate binding to calcium ions, resulting in the loosening of the hydrogel network structure and ultimately leading to a decrease in G′ [15,16].

Comparing AC to ALP samples with the same alginate concentrations, the cultured beads exhibited higher G′ than that in the uncultured samples. There were mainly two reasons for this variation: firstly, the proliferation of *L. plantarum* filled the inner space of the hydrogel beads, forming microcolonies that can enhance G′. Secondly, bacterial growth was accompanied by the metabolism of acids. The charge density of hydrogel beads increased with the decrease in pH, thereby decreasing chain flexibility, shortening the distance between molecular chains. Eventually, the number of interactions and entanglements between alginate molecules would increase, leading to the rising in G′. Similar results were also reported by Bastos et al. [21], who studied the interaction between chitosan and whey proteins by isothermal titration calorimetry (ITC) and turbidity measurements; they found that with the decrease of pH, the charge density and molecular size of chitosan increased corresponding with the decreasing of chain flexibility leading to the increasing in G′.

The utilization of dynamic viscosity and imaginary viscosity can effectively characterize the viscoelastic properties of polymer systems. For all the samples, with the increasing in frequency, both dynamic viscosity and imaginary viscosity decreased, indicating shear-thinning behavior [22]. The shear-thinning behavior stems from the change in the macromolecular organization of the hydrogel beads with variations in frequency [23]. At low frequencies, the breakage rate of intermolecular entanglement between alginate chains caused by the applied force was balanced by the breakage rate of newly formed entanglements. At higher frequencies, fragmentation dominated the formation of new entanglements and molecules were aligned in the direction of force application, leading to the decrease of viscosity with the increasing shear rate.

When comparing the difference between imaginary viscosities of AC and ALP groups, we found that the absolute slope values of η″ curves in the ALP group increased to different degrees compared to those in the AC group, which originated from the electrostatic interactions between the polysaccharide chains [22]. During the cultivation process, the ALP samples were exposed to a low-pH environment, resulting in an increased charge density of the hydrogel beads. The reduction in intermolecular distance and increase in electrostatic interactions between the polysaccharide chains would thus lead to a greater contribution of elasticity within the hydrogel beads of the ALP groups, which also coincided well with the results in Figure 5a,b.

### 3.5. In Vitro Simulation of Gastrointestinal Digestion

**Figure 6:**
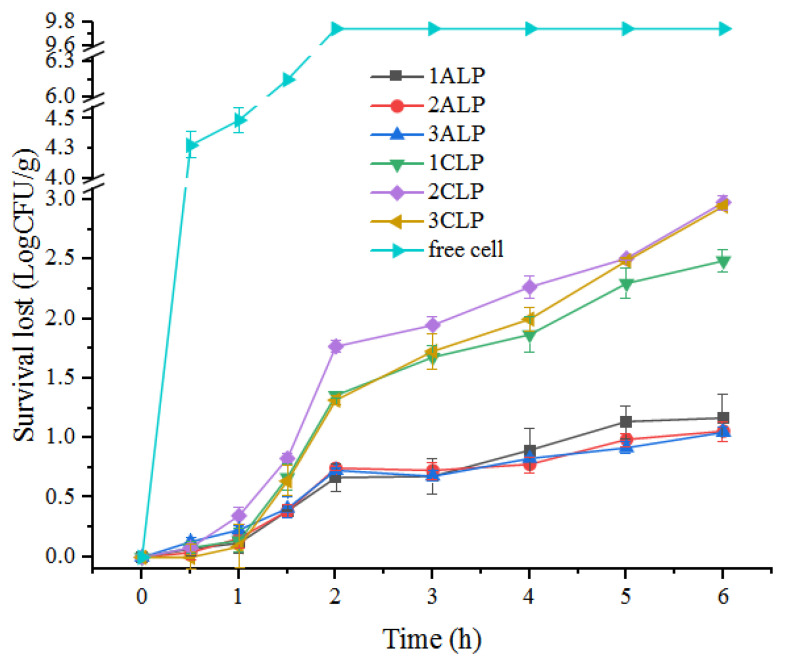
In vitro simulation of gastrointestinal digestion of different probiotic hydrogel beads.

The gastrointestinal digestion properties of hydrogel beads fabricated using in situ co-culturing (ALP) and traditional method (CLP) were shown in Figure 6. Free cells lost all the viability within 2 h of gastric digestion. After microencapsulation, for ALP samples, cell viability lost 1.09 Log CFU/g during the entire gastrointestinal digestion; however, for CLP samples, cell viability lost around 2.81 Log CFU/g, which was nearly three times higher than that in ALP samples. Considering the higher initial loading viable cells in ALP samples (10.65 ± 0.09 Log CFU/g) as compared with that in CLP samples (9.88 ± 0.09 Log CFU/g), a total 2.44 Log CFU/g improvement was found after the entire gastrointestinal digestion. Moreover, unlike the CLP samples, which have higher survival rates with the increase of alginate concentration, no significant difference was found in ALP samples with different alginate concentrations. The great improvements in viabilities of cells in ALP samples can be due to two main reasons: on one hand, the dense micro colonies inside the beads can not only serve as filling agents spread over the crosslinked alginate molecular chains that enhanced the structure strength of hydrogel beads (Figure 5) and defend the erosion of gastric fluid to probiotic cells; on the other hand, the dense growth of cells in clustered microcolonies would facilitate strong communication between probiotics, which in turn triggered quorum sensing and generated resistance to external harsh conditions. As reported by Philip [24], the microcolonies generated by in situ cultivation were in a highly protected phenotype state that resembled spore-like cell differentiation. In high-density microcolony forms, bacteria would produce signaling molecules that activate regulatory factors of target genes at a threshold concentration, thus achieving regulation of quorum sensing, allowing microcolonies generated by in situ cultivation to withstand external damage by harsh conditions.

## 4. Conclusions

The current study demonstrated that probiotic microcapsules fabricated by an in situ cultivation method could not only increase the loading capacity of viable cells after encapsulation, but also greatly improve the survival of cells during gastrointestinal digestion. Up to 10.34 ± 0.02 Log CFU/g of viable cell concentration was achieved after 24 h of cultivation, and only a loss of 1.09 Log CFU/g of viable bacteria was observed after digestion in the gastrointestinal tract, resulting in an increase of approximately 2.44 Log CFU/g after the end of digestive process. The method developed in this research holds potential for application to a broad range of probiotics, and further investigation and optimization of this method may lead to commercial products.

## Figures and Tables

**Table 1:** The composition and preparation of hydrogel bead samples.

Group	Sample Name	Sodium Alginate (wt%)	Concentration of Probiotics in the Sample (LogCFU/g)	Incubation Time (h)
Before incubation Non-bacteria group	1BC	1	/	/
	2BC	2		
	3BC	3		
Before incubation Contains low concentration of bacteria group	1BLP	1	6	/
	2BLP	2		
	3BLP	3		
After incubation Non-bacteria group	1AC	1	/	24
	2AC	2		
	3AC	3		
After incubation Bacteria-containing group	1ALP	1	Change with time	24
	2ALP	2		
	3ALP	3		
Control group	1CLP	1	9	/
	2CLP	2		
	3CLP	3		

**Table 2:** Particle sizes of different hydrogel beads.

	Before Incubation		After Incubation	
	Name	Diameter (mm)	Name	Diameter (mm)
Non-bacteria group	1BC	2.109 ± 0.035 ^h^	1AC	2.458 ± 0.069 ^f^
	2BC	2.466 ± 0.078 ^f^	2AC	2.617 ± 0.034 ^e^
	3BC	2.787 ± 0.051 ^c^	3AC	2.867 ± 0.055 ^b^
Bacteria-containing group	1BLP	2.111 ± 0.096 ^h^	1ALP	2.358 ± 0.129 ^g^
	2BLP	2.69 ± 0.036 ^d^	2ALP	2.846 ± 0.065 ^bc^
	3BLP	2.644 ± 0.046 ^de^	3ALP	3.024 ± 0.047 ^a^

Numbers with different superscripts (a–h) are significantly different (*p* < 0.05).

## Data Availability

The data presented in this study are available on request from the corresponding author.
